# Endoscopic ultrasound-guided hepaticogastrostomy stent exchange using a novel spiral plastic stent

**DOI:** 10.1055/a-2307-7866

**Published:** 2024-05-17

**Authors:** Takeshi Ogura, Atsushi Okuda, Saori Ueno, Nobu Nishioka, Hiroki Nishikawa

**Affiliations:** 1 Endoscopy Center, Osaka Medical and Pharmaceutical University Hospital, Takatsuki, Japan; 2 2nd Department of Internal Medicine, Osaka Medical and Pharmaceutical University, Takatsuki, Japan

Endoscopic ultrasound-guided hepaticogastrostomy (EUS-HGS) can be attempted for patients with failed endoscopic retrograde cholangiopancreatography 1 2 . EUS-HGS has recently become indicated for not only malignant biliary obstruction, but also benign biliary disease 3 4 . In benign biliary disease, a self-expandable metal stent (SEMS) might not be suitable, because stent removal may be challenging if the duration of stent deployment is prolonged. Therefore, a plastic stent is usually used. According to a previous study, regular stent exchange may have a clinical impact on prevention of benign biliary disease 4 . However, flaps are provided at the distal end in most plastic stents to prevent stent dislocation. Unlike the common bile duct, the intrahepatic bile duct has side branches; therefore, during stent removal, the flaps may become stuck in the side branches. Consequently, a plastic stent with flaps has a risk of rupture, as previously described 5 .

To improve stent removability, a spiral-shaped plastic stent has been designed and is available in Japan (Gadelius Medical Co., Ltd, Tokyo, Japan) ( Fig. 1 ). This stent has no side flaps; therefore, stent removal through EUS-HGS can be easily performed without the stent flaps becoming stuck in the bile duct side branches. To prevent stent dislocation, the distal end is spiral in shape, and to prevent stent migration, the proximal end is a pigtail shape. A case of successful EUS-HGS stent exchange using this novel plastic stent is reported.

**Fig. 1:**
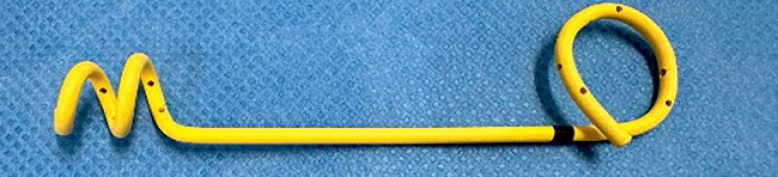
A spiral-shaped plastic stent (Gadelius Medical Co., Ltd, Tokyo, Japan).

An 88-year-old woman was admitted because of obstructive jaundice caused by a hepaticojejunostomy stricture. In addition, a huge intrahepatic bile duct stone was present. She first underwent EUS-HGS using SEMS, and because of her advanced age, only stent exchange to a spiral-shaped plastic stent was performed ( Fig. 2 ). After 6 months, she was admitted again for stent exchange. First, a standard duodenoscope was inserted into the stomach. Then, after a guidewire was deployed beside the stent ( Fig. 3 ), stent removal using grasping forceps was attempted. As the stent had no side flaps, stent removal was easily performed without stent rupture ( Fig. 4 ). After evaluation of the biliary system, a spiral-shaped plastic stent was deployed without any adverse events ( Fig. 5 , Video 1 ).

**Fig. 2:**
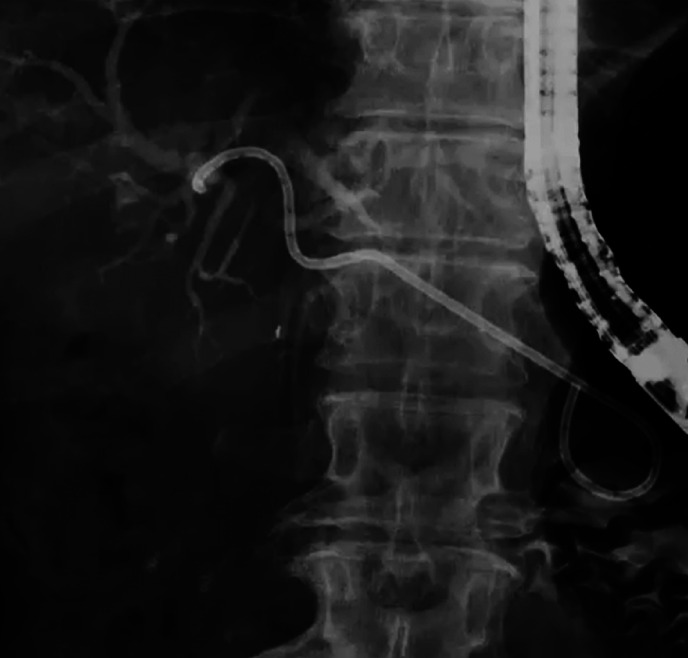
The spiral-shaped plastic stent was deployed as an endoscopic ultrasound-guided hepaticogastrostomy stent.

**Fig. 3:**
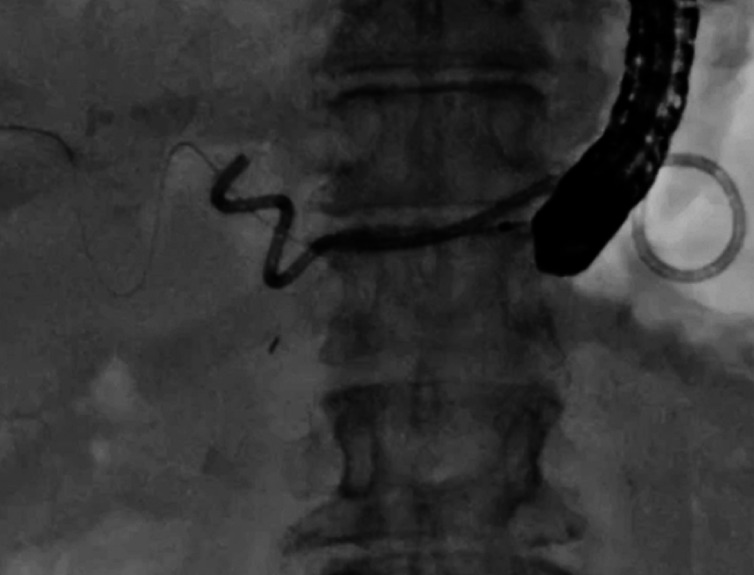
A guidewire was deployed beside the stent.

**Fig. 4:**
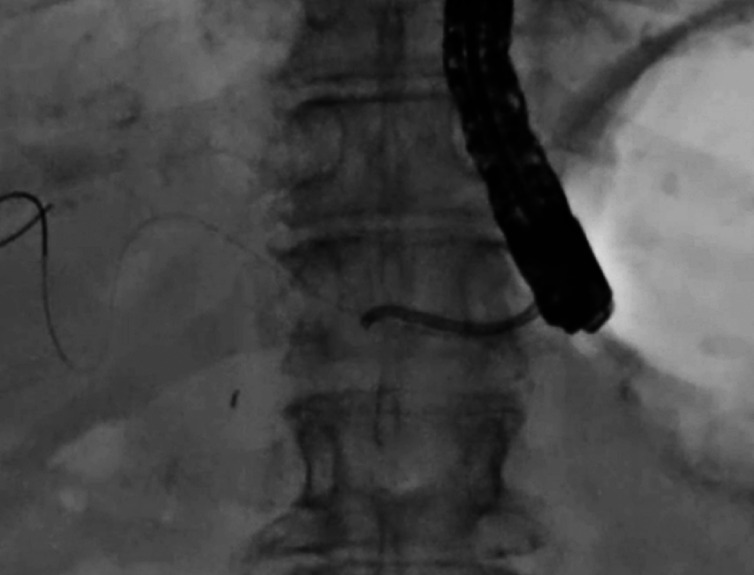
As the stent had no side flaps, stent removal was easily performed without stent rupture.

**Fig. 5:**
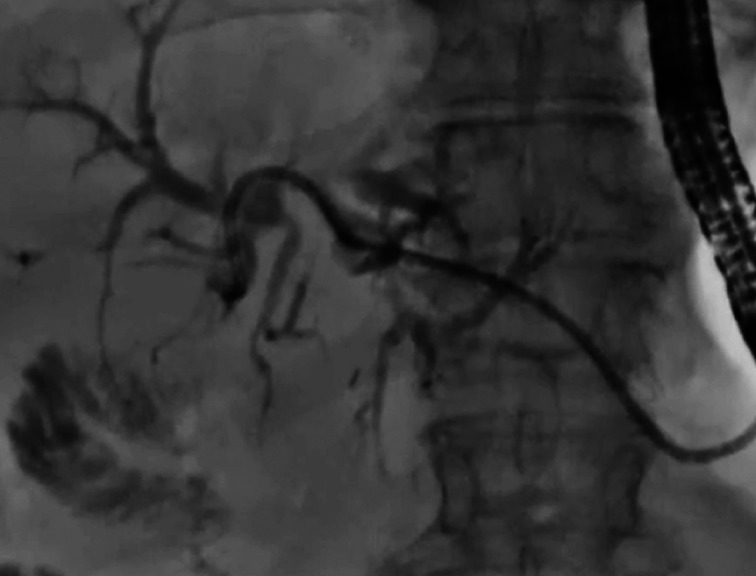
The spiral-shaped plastic stent was deployed.

Removal of an endoscopic ultrasound-guided hepaticogastrostomy stent was attempted. As this stent had no side flaps, removal was easily performed without stent rupture.Video 1

In conclusion, a spiral-shaped plastic stent may be useful as an EUS-HGS stent for patients who undergo scheduled stent exchange because of its easy removability.

Endoscopy_UCTN_Code_TTT_1AS_2AH

## Notes

Endoscopy E-Videos https://eref.thieme.de/e-videos
*E-Videos* is an open access online section of the journal *Endoscopy* , reporting on interesting cases and new techniques in gastroenterological endoscopy. All papers include a high-quality video and are published with a Creative Commons CC-BY license. Endoscopy E-Videos qualify for HINARI discounts and waivers and eligibility is automatically checked during the submission process. We grant 100% waivers to articles whose corresponding authors are based in Group A countries and 50% waivers to those who are based in Group B countries as classified by Research4Life (see: https://www.research4life.org/access/eligibility/ ). This section has its own submission website at https://mc.manuscriptcentral.com/e-videos .
